# Spectrum of Darkness, Agent of Light: Myopia, Keratoconus, Ocular Surface Disease, and Evidence for a Profoundly Vitamin D-dependent Eye

**DOI:** 10.7759/cureus.2744

**Published:** 2018-06-05

**Authors:** James McMillan

**Affiliations:** 1 Ophthalmology, Medina Eye Ps

**Keywords:** vitamin d insufficiency, vitamin d3, myopia, keratoconus, glaucoma, macular degeneration, dry eyes, developmental biology, astigmatism, cornea

## Abstract

Serial observations obtained over more than eight years and 10,000 patient encounters in a general ophthalmology practice serving a population highly prone to chronic vitamin D (D3) deficiency, facilitated by the Oculus Pentacam Scheimpflug imaging system (Oculus Optikgeräte GmbH, Wetzlar, Germany), resulted in the recognition of consistent, predictable, and highly reproducible patterns of mechanical, optical, and physiologic change in the cornea and other ocular structures correlated to adequate vs. inadequate vitamin D availability. These patterns were identified from an analysis of more than 20,000 topographical and digital imaging studies, manifest refraction results, and other clinical ophthalmic exam findings recorded during patient visits. The main outcome measures included improved corneal and global optical quality and function, decreased ametropia, improved stability, and decreased subjective symptoms of compromised acuity and comfort. Adequate D3 replacement consistently yielded some degree of objective structural improvement in all subjects observed. The rate of improvement varied and synergistic interaction with cofactors was also suggested in particular topical steroids. A plausible explanation for the cause and mechanism of most myopia emerged and keratoconus, in particular, appears to be the extreme presentation of otherwise common corneal disturbances associated with inadequate vitamin D availability. Emmetropization mechanisms appear to awaken and reactivate with adequate D3. Intraocular pressure control likewise shows evidence of being vitamin D regulated and may play a significant interactive role in emmetropization and relief from ametropia. Ocular surface disease and inflammatory activity can be markedly alleviated in addition. As the findings are most readily appreciated via topographical map changes, a series of case reports are presented, selected from the mass of similar data, to illustrate specific aspects of these findings in the hope of inspiring controlled trials to better delineate their significance. Taken as a whole, these observations suggest the human eye may be profoundly dependent upon adequate vitamin D availability for many critical optical, structural, and physiologic properties. Myopia may represent the end result of adverse emmetropization feedback generated by low vitamin D-related irregular corneal astigmatism.

## Introduction

There is growing global interest in the physiologic effects of vitamin D (D3) in all its forms. From an ocular standpoint, the role in macular and retinal health had been advanced, as well as the involvement in corneal inflammatory response, wound healing, and dry eye disease [1-2]. There has also been a suspicion that deficiency is a risk factor for myopia [3-5] though a recent report appears to contradict this being a direct effect [6]. Vitamin D receptor (VDR) is expressed in the cornea, ciliary body, lens, retina, and retinal pigment epithelium and polymorphisms in the receptor and its start codon have been linked to myopia [7-9].

In this report, observations drawn from over 10,000 patient encounters, representing a cohort of more than 2000 individuals followed by one physician at a single location and collected over more than eight years, provide compelling evidence for the rapid and apparently certain influence of vitamin D3 on multiple aspects of ocular physiology and function. Corneal optical quality and development, ocular structural integrity and maintenance, intraocular pressure modulation and measurement, and immunological behavior pertaining to dry eye and ocular surface disease are beneficially impacted clinically. In addition, accumulating evidence supports a desirable influence on cataract-associated symptoms. Taken as a whole, they present a novel interpretation of the eye as profoundly dependent on vitamin D and, in the process, suggest compelling hypotheses for the mechanisms of both myopia and keratoconus development. In particular, the implied biomechanical model readily applies to the growing understanding of feedback-driven maturational emmetropization and offers a feasible explanation for the global surge in myopia. Keratoconus, in particular, appears to represent one extreme of an otherwise common continuum. The findings of keratoconus and other forms of keratoectasia, such as may follow refractive surgery, can be significantly and reliably improved by adequate vitamin D availability.

These discoveries were facilitated by the availability of the Oculus Pentacam Scheimpflug imaging system (Oculus Optikgeräte GmbH, Wetzlar, Germany). The ability to efficiently provide precise and reproducible corneal images, simultaneous thickness determination, internal and external curvature data, and density quantification of translucent structures revealed the salient characteristics in a chronically D3 deficient population. Remarkably, corneal shape improvement and its optical influence can be objectively demonstrated within less than one week of instituting effective supplementation. Topographical response has remarkably proven to be literally 100% predictable in over 20,000 scans obtained in this population. The improvement trend continues over time, as long as serum 25(OH)D3 level is adequately maintained. Moreover, benefits may be amplified via synergistic interaction with topical corticosteroids, accelerating improvement in corneal shape and optics, substantially benefiting the control of ocular surface disease and dry eye symptoms, while, in addition, suggesting the suppression of the intraocular pressure “steroid response” in susceptible individuals. The following cases were chosen to illustrate multiple aspects of this discovery and will hopefully stimulate additional research.

## Materials and methods

Corneal data from in excess of 12,000 Pentacam scans, representing more than 2000 individuals, were obtained during office visits to a single general ophthalmology practice between 2008 and 2016. The Pentacam has been established as a reliable tool for the evaluation of corneal ectasia and other refractive and structural concerns of the anterior segment. Previously validated analyses employing this instrument include corneal clarity, via the quantification of reflectance, true net power from simultaneous anterior and posterior surface curvature measurements, true elevation data, and keratoconus grading [10]. The system allows for a ready analysis of change over time, whether topographical or in regard to other parameters, such as corneal thickness, with options to display multiple sequential examinations and calculate and map interval change. “True net power,” incorporating total corneal refractive power from both internal and external surface contributions is particularly useful. Scans were reviewed and interpreted by the same physician at each visit and compared serially over time. In most cases, comprehensive examination findings were available on at least an annual basis to provide concurrent, corresponding data for visual acuity, intraocular pressure, lens changes, and retinal status.

The data were gathered in Bellevue, Washington State, USA. The latitude (47ᵒ 37’ N) and local weather patterns markedly limit potential sun exposure and natural vitamin D production. The surrounding area in Western Washington laid claim to all of the top 15 of the 101 cities with the lowest average sunshine in the continental United States. Bellevue was ranked sixth [11]. The majority of the regional population is usually found to be chronically vitamin D deficient in the absence of supplementation [12]. Serum 25(OH)D3 testing in the non-supplemented population typically yields values between 10 and 24 ng/cc, but levels as low as 3 ng/cc have been encountered among the patient population observed (anecdotal). This setting offers an ideal environment for the identification of vitamin D influence upon the eye.

The risks and benefits, known and speculated, of the intake of Vitamin D supplements were routinely discussed in depth with all patients and, where applicable, their parent/guardian. They were free to choose to pursue the recommended intake or not, according to their own informed consent or that of their parent/guardian. Only over-the-counter available D3 forms were involved, representing a wide array of brands and delivery options (capsules, gelcaps, drops, and “gummies” or chewables). A starting dose of 5000 IU per day was typically recommended for those aged 10 or older. For those under the age of 10 or of light build, 1000 IU per 25 pounds body weight per day was recommended. For all patients, with or without supplementation, an annual testing of the serum 25(OH)D3 level was encouraged, and for any individuals desiring to take 10,000 IU per day or more on an extended basis, testing was considered necessary within one to three months of starting such a regimen. A limitation of this study is a lack of a specific baseline and post-supplementation serum 25(OH)D3 levels for all patients. Recent insurance and clinical guideline restrictions for the testing of serum vitamin D impede readily obtaining levels in the absence of a limited number of approved diagnoses, so results are correlated to the amount of supplementation in most cases. All record keeping, storage, communications, and office interactions were conducted in Health Insurance Portability and Accountability Act (HIPPA)-compliant fashion. Vitamin D and the topical steroid preparations used were kept within established safe or approved ranges, and serum 25(OH)D3 levels monitored as necessary to maintain them within the commonly utilized normal range of 30-100 ng/cc. The University of Washington Human Subjects Division (Institutional Review Board) was consulted once the desirability of presenting this case data became evident. Their determination was that no additional review was required. All aspects of this work adhered to the tenets of the Declaration of Helsinki.

## Results

Identical twins with differing natural sunlight exposure

**Figure 1:**
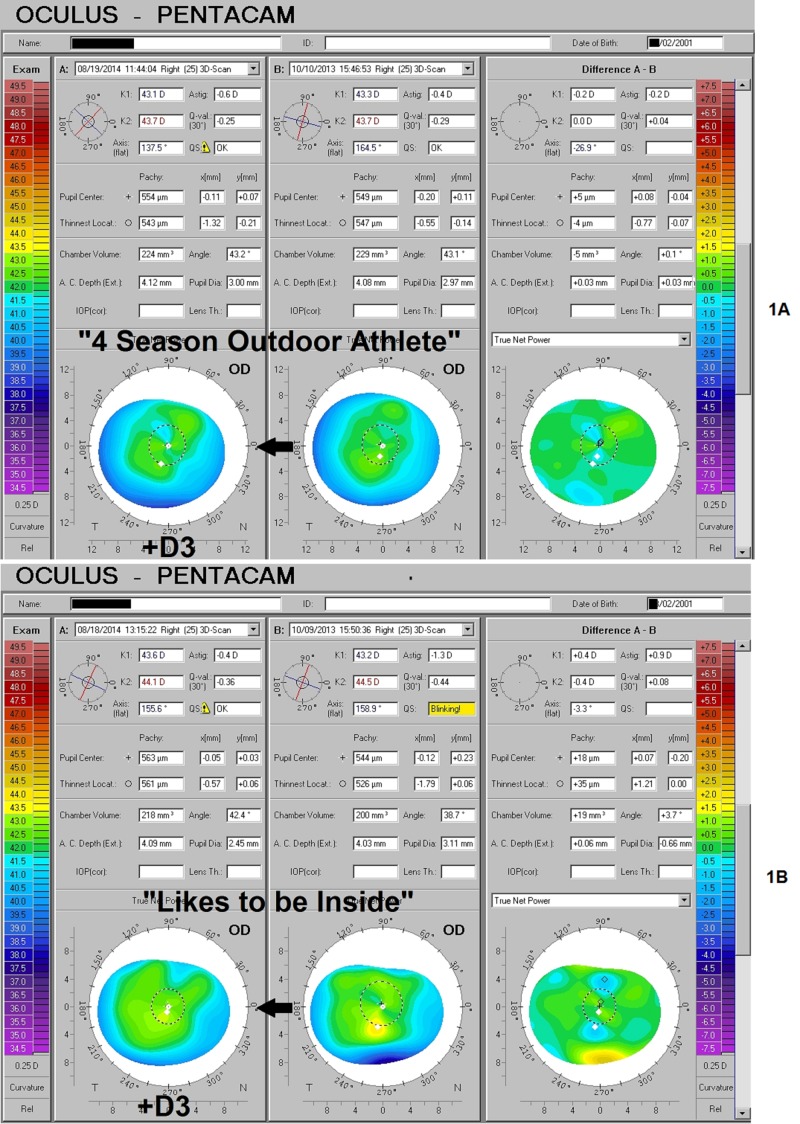
Identical Twins - Outdoor vs. Indoor Preference, Before and After D3 Serial observations of the corneal and refractive impact of relative differences in natural sunlight exposure, as well as subsequent D3 supplementation in monozygotic twins during peak growth years. A) Mild irregular astigmatism further diminishes in the right eye of a "four season outdoor athlete," who at baseline had such sun exposure as might be obtained in the Pacific Northwest. B) Her sister, who preferred being inside reading or on the computer, had considerably more irregular astigmatism with typical "inferior-steep distribution" at the outset, which was reduced even more. The "delta" or net change, obtained via the subtraction of the older values from the new, is depicted in the maps to the right ("Difference A - B"). Low areas are seen to rise and high areas to fall exactly as required to create a more uniform and symmetrical form overall, approaching the ideal aspheric shape having the highest power at the axis and gentle concentric flattening toward the periphery.

A pair of 12-year-old, monozygotic twins presented for baseline examinations. One, a self-described four-season outdoor athlete, had no complaints and possessed uncorrected acuity of 20/20 in each eye and manifest refractions of the right eye (OD) +0.50 -0.25 x 85 and left eye (OS) +0.75 -0.25 x 93. Pentacam imaging revealed a very mild axial irregular astigmatism pattern, with an inferior-steep predominance (Figure 1A). Her sister, who preferred being indoors pursuing computer work and reading, was, by contrast, recently noticing a difficulty with her distance acuity (OD 20/20-1 and OS 20/25). Her manifest refractions were OD plano -0.50 x 83 and OS +0.75 -0.50 x 88. The Pentacam imaging also demonstrated irregular corneal asymmetry of the same general nature as that of her sister but substantially more pronounced (Figure 1B). Over the following year, both of the twins supplemented vitamin D3 at 2,000 to 3,000 IU per day (“frequently,” per their mother). At the follow-up examination, now age 13, neither reported any relative change in their time spent outdoors; however, decreased irregular astigmatism was found in all eyes and manifest refractions had converged on emmetropia: the "outdoor" twin: OD +0.50 – 0.75 x 85, OS +0.50 – 0.75 x 85, and her "indoor" sibling: OD plano -0.25 x 95, OS +0.25 – 0.25 x 90 (Figures 1A-1B). While the refractive shifts are modest, correlation with topographical changes showing improved symmetry about the point of gaze and trend toward an idealized aspheric form is consistent with significant supplemental impact in both cases. The baseline studies furthermore support a differential effect of daylight exposure and presumed increased UVB-mediated vitamin D production upon the corneal shape and optical behavior in genetically identical individuals with otherwise similar environmental influences.

Teens with and without adequate vitamin D availability

**Figure 2:**
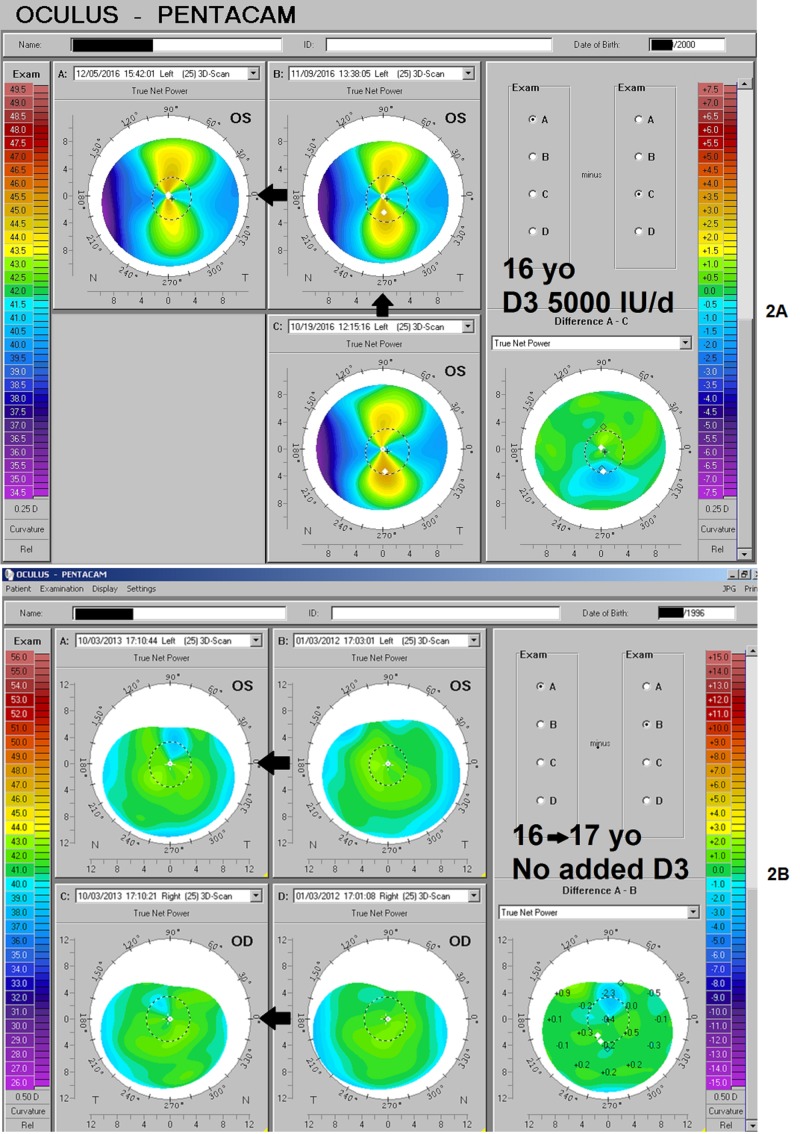
Teen Receiving D3 vs. Same Age with no Supplementation Figure 2A shows the change in corneal true net power mapping over two months for the left eye of a 16-year-old girl, as astigmatic distortion reduces in the presence of supplemental vitamin D3. A more symmetrical pattern results. Figure 2B shows the deterioration of corneal symmetry over the same age range in a boy with no supplementation. The increased asymmetry demonstrates the same downward displacement ("inferior-steep") pattern that diminished with adequate vitamin D3.

Over the course of two months, a 16-year-old girl taking 5000 IU/day vitamin D3 at the urging of her mother demonstrated a typical reduction in irregular corneal astigmatism/asymmetry, from the initially pronounced "inferior-steep" pattern most commonly observed in vitamin D deficient individuals, to an overall lower amount of distortion of all kinds—by then much more symmetric about the mid-point (Figure 2A). In contrast, over a two-year period, a boy of similar age demonstrated a shift toward a more irregular and "inferior-steep" pattern in both eyes (Figure 2B).

Siblings with suppression of myopic progression

A 13-year-old girl had baseline developmental myopia, with manifest refractions OD of -0.50 – 0.25 x 90 and OS of -0.25 – 0.25 x 59. Over the ensuing two years, serial Pentacam findings demonstrated the bilateral development of focal, peri-axial corneal steepening and a gradual rise in right eye manifest refraction at age 15: OD -1.50 -0.25 x 93 and OS -1.00 – 0.25 x 50. Oral vitamin D3 intake of 2000 IU/day was started. Seventeen months later, at age 17, despite passage through the period of peak growth, the manifest refraction of the right eye declined slightly to -1.50 sphere and the left demonstrated stability, at -1.25 sphere. Pentacam imaging revealed a reduction in the focal, peri-axial astigmatic irregularity, and steep regions in both eyes.

A 10-year-old boy had baseline developmental myopia, with manifest refraction OD -0.50 – 0.25 x 88 and OS -0.50 – 0.25 x 177. Over the ensuing two years, serial Pentacam findings demonstrated the bilateral development of focal, peri-axial corneal steepening, and a gradual rise in myopia to OD manifest refraction -1.25 -0.25 x 85 and OS -1.25 sphere. Oral vitamin D3 intake of 2000 IU/day was started. Seven months later, at age 13, despite entering the peak growth period, the manifest refraction OD declined slightly to -1.25 – 0.25 x 85 and the OS remained stable at -1.25 sphere. Both eyes demonstrated a reduction in peri-axial astigmatic irregularity and steep regions.

Dry eye disease

**Figure 3:**
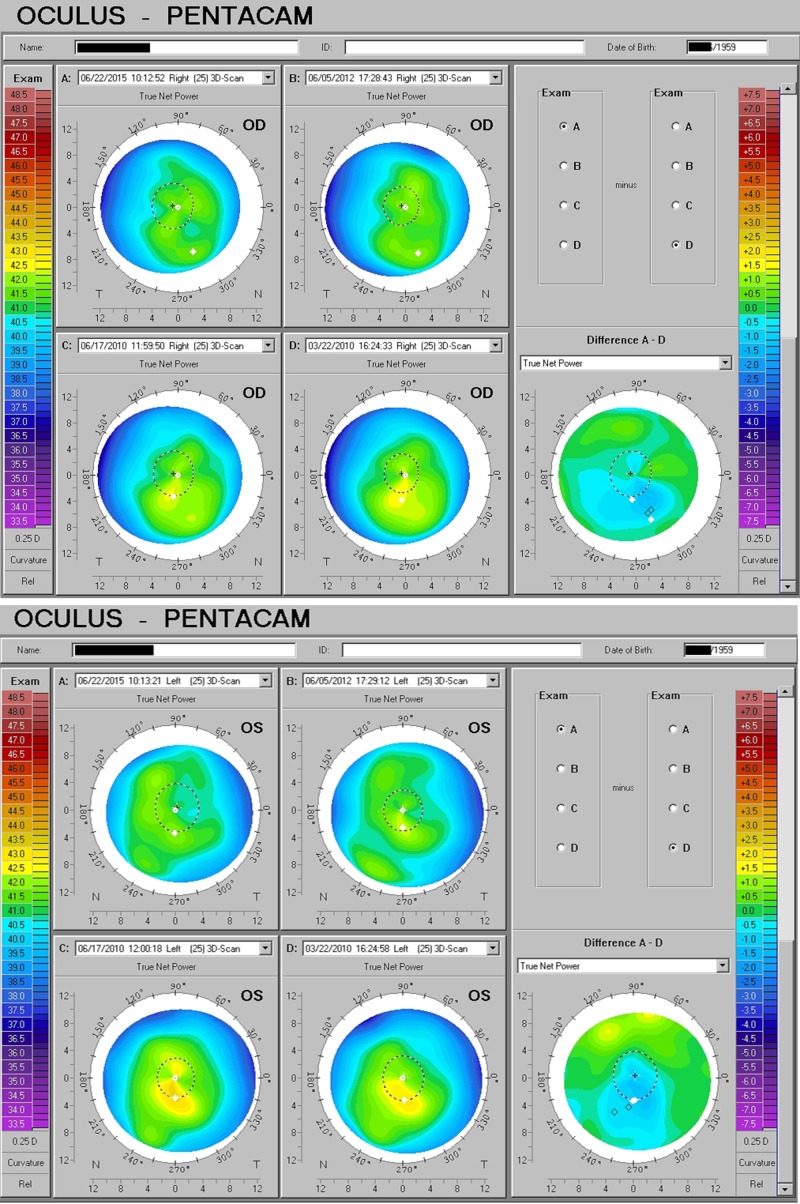
Dry Eye Response to D3 Progressive structural and optical improvement in response to adequate D3 supplementation over a period of five years, in a patient with initially disabling dry eye complaints. The physical changes accompanied his decreasing symptoms and improving acuity.

A 51-year-old man presented for an additional opinion for “dry eyes,” reporting he had been told his corneas “looked like golf balls.” Attempts had been made to treat him with teardrops, topical antibiotics, systemic doxycycline, and topical steroid drops. None of the interventions had been significantly helpful for the control of his symptoms. Baseline serum 25(OH)D3 was determined to be 26.7 ng/cc. After discussing options, he elected to pursue D3 replacement at 50,000 IU/week for four weeks, followed by 4000 IU/day. After one month, he was additionally treated with a course of topical difluprednate (Durezol) BID for one week, then QD for one week, accompanied by topical azithromycin (Azasite) according to the same schedule. Repeat blood testing eight months later found the 25(OH)D3 at 88 ng/cc and the patient asymptomatic, on no artificial tears, and able to again take up successful soft contact lens wear. Pentacam analysis confirmed a markedly reduced irregular astigmatism (Figure 3).

Age-related macular degeneration (AMD) concern

**Figure 4:**
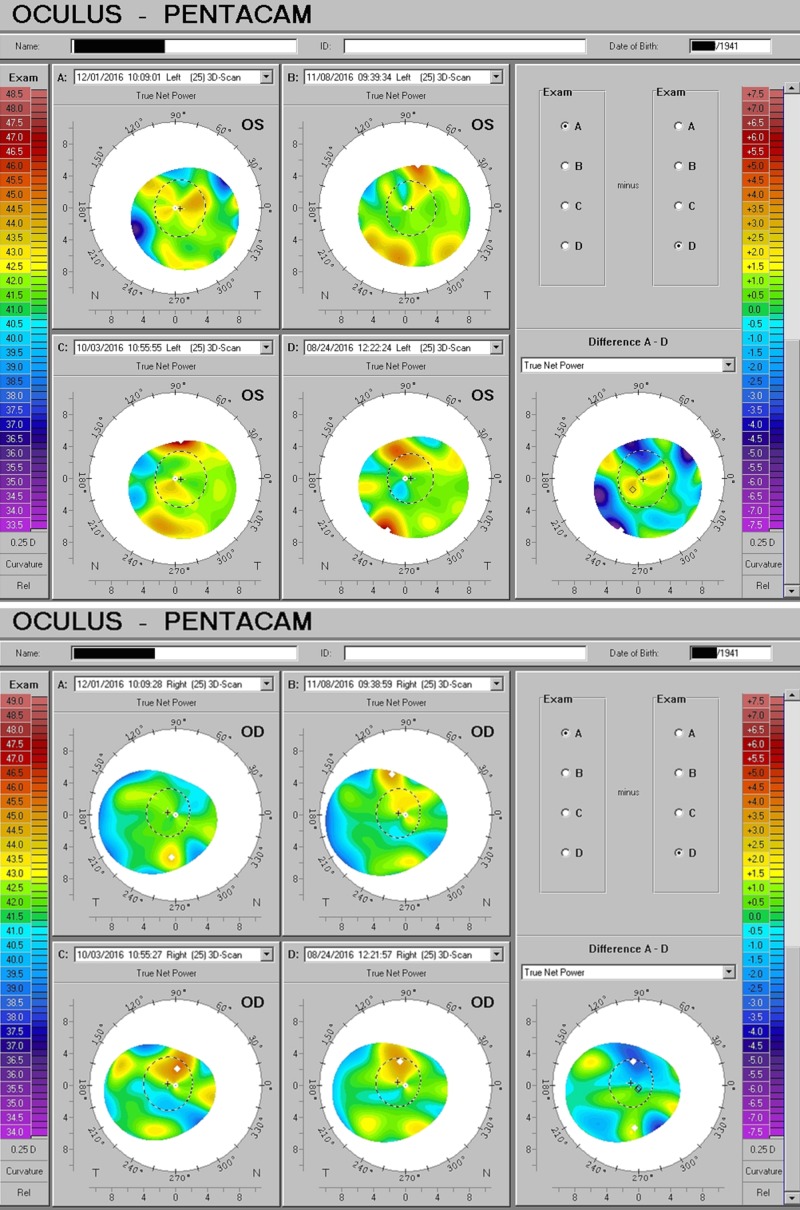
AMD Acuity Concern Addressed with D3 Visual impairment in patient diagnosed with age-related macular degeneration markedly improved with the corneal optical response accompanying D3 supplementation. AMD: age-related macular degeneration

A 75-year-old man seeking an additional opinion as to whether his declining vision was caused by progressive macular degeneration—known to have considerable macular drusen and retinal pigment epithelial disruption—was found to have substantial irregular corneal astigmatism in addition. He had already undergone successful cataract surgery years earlier. At presentation, visual acuity was measured at OD 20/25 with effort, OS 20/30 with effort. He was started on vitamin D3 supplementation at 5000 IU/day and when seen in follow-up two months later, remarked “significant improvement!” Four months after starting the supplementation, visual acuity was correctable to 20/20 in each eye and corneal optical quality much improved (Figure 4).

Keratoconus

**Figure 5:**
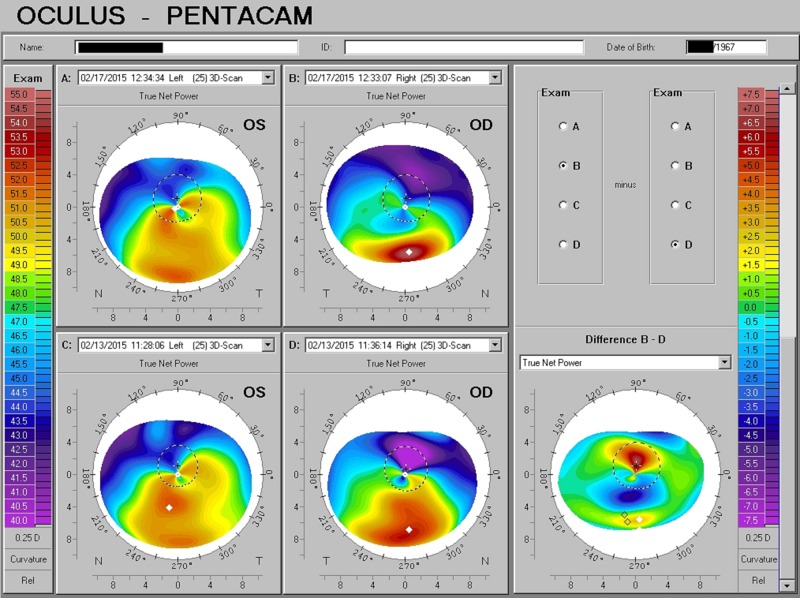
Severe Keratoconus with Rapid Response to D3 Significant improvement in corneal form and optical function in less than a week with adequate D3 supplementation.

A 47-year-old woman, a resident of the Southeast visiting Bellevue for one week, was encouraged by her parents to seek additional opinion for her complaint of a rapid decline in vision, diagnosed by another physician as due to keratoconus. She could no longer wear contact lenses, could not pass the visual examination for driving, and was concerned that her reading was so difficult that she might not be able to continue work. She had been advised to consider bilateral penetrating keratoplasty. At presentation, the best spectacle-corrected acuity was OD 20/80 and OS 20/50, with a complaint of multiple overlapping and indistinct images. Following a discussion and given her acute need to determine a course of action, she elected to start 10,000 IU/day of D3. In addition, prednisolone acetate drops were prescribed BID for one week and then QD for one week. She was reassessed just four days later and dramatic improvement was already demonstrated: the best-corrected acuity was now OD 20/40 and OS 20/30, with a topographical confirmation of substantial beneficial change (Figure 5). Upon her return home, she resumed her employment and reported continued improvement in her symptoms.

**Figure 6:**
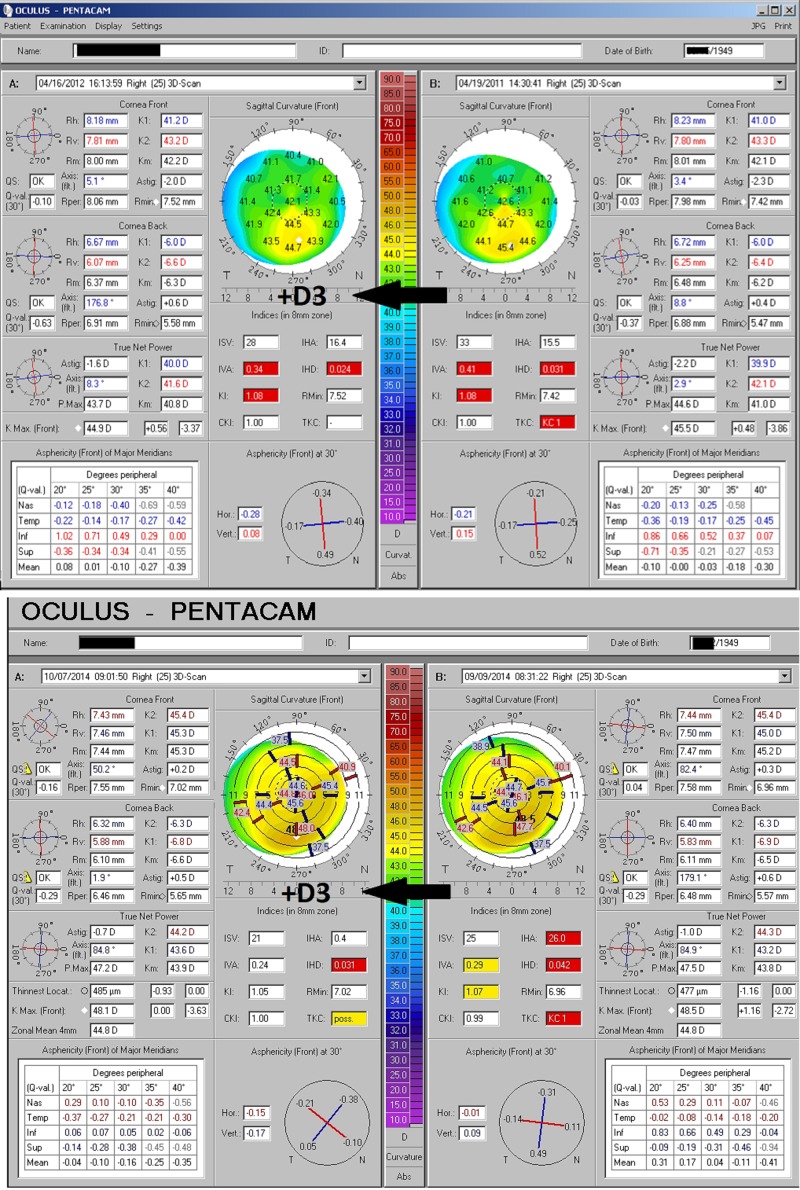
Additional Keratoconus Improvement with D3 Two more examples of the reversal of keratoconus findings and grading with supplementation of adequate D3 and without the addition of corticosteroids. The Pentacam analysis includes topographical mapping and a variety of parameters related to front and back surface symmetry and curvature characteristics to generate indices comparable to a database of normal corneas. Where indices fall significantly outside normal expectations, values are flagged as borderline (yellow) or abnormal (red). A combination of abnormal indices will lead to designation as likely keratoconus, graded on a scale from one to four (KC1, KC2, etc.). Both individuals demonstrate a reduction in flagged indices and keratoconic grading.

Two additional examples of patients with keratoconus, as determined by a Pentacam topometric analysis of multiple indicators, demonstrate a similar response to adequate D3. Both individuals were coincidentally born in 1949 (Figure 6). In neither case were steroids applied; the changes correlating to increased vitamin D intake alone.

Intraocular pressure response

**Figure 7:**
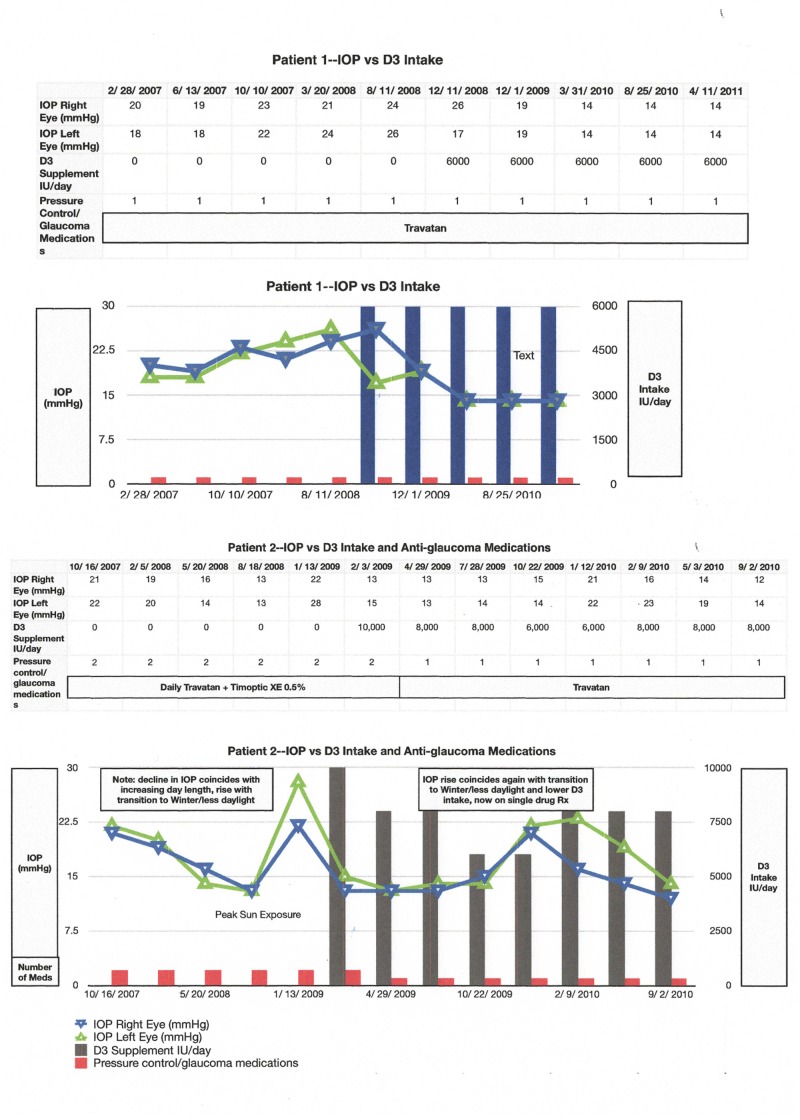
D3 Intraocular Pressure Impact Two examples of glaucoma patients under treatment, demonstrating a reduction of intraocular pressure and/or the need for medication following adequate D3 supplementation.

Two individuals with a longstanding glaucoma diagnosis were under treatment with topical medication. In the second example, serial measurements suggest both a dose-response phenomenon and evidence for challenge/re-challenge (Figure 7).

Post-LASIK irregular astigmatism

**Figure 8:**
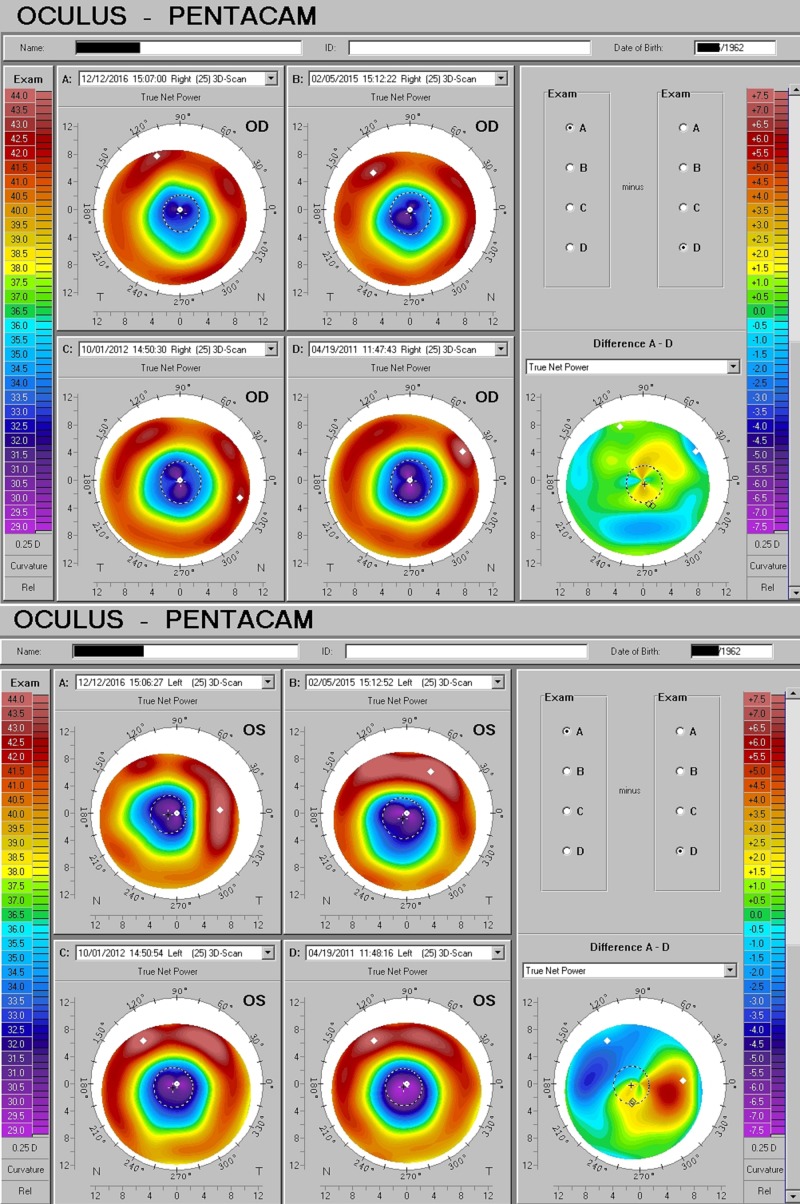
Post-LASIK Distortion Response to D3 Improving corneal optics in both eyes of a patient with considerable post-LASIK irregular astigmatism. LASIK: laser-assisted in-situ keratomileusis

A 54-year-old woman with a qualitatively limited, best spectacle-corrected acuity following laser-assisted in-situ keratomileusis (LASIK), affecting both eyes, complained of poor acuity, glare, and, in particular, poor night vision. Symptoms slowly resolved following D3 replacement at 5000 IU/day over a period of five years (Figure 8).

Dose response: 10,000 IU/day vs. 5000 IU/day

**Figure 9:**
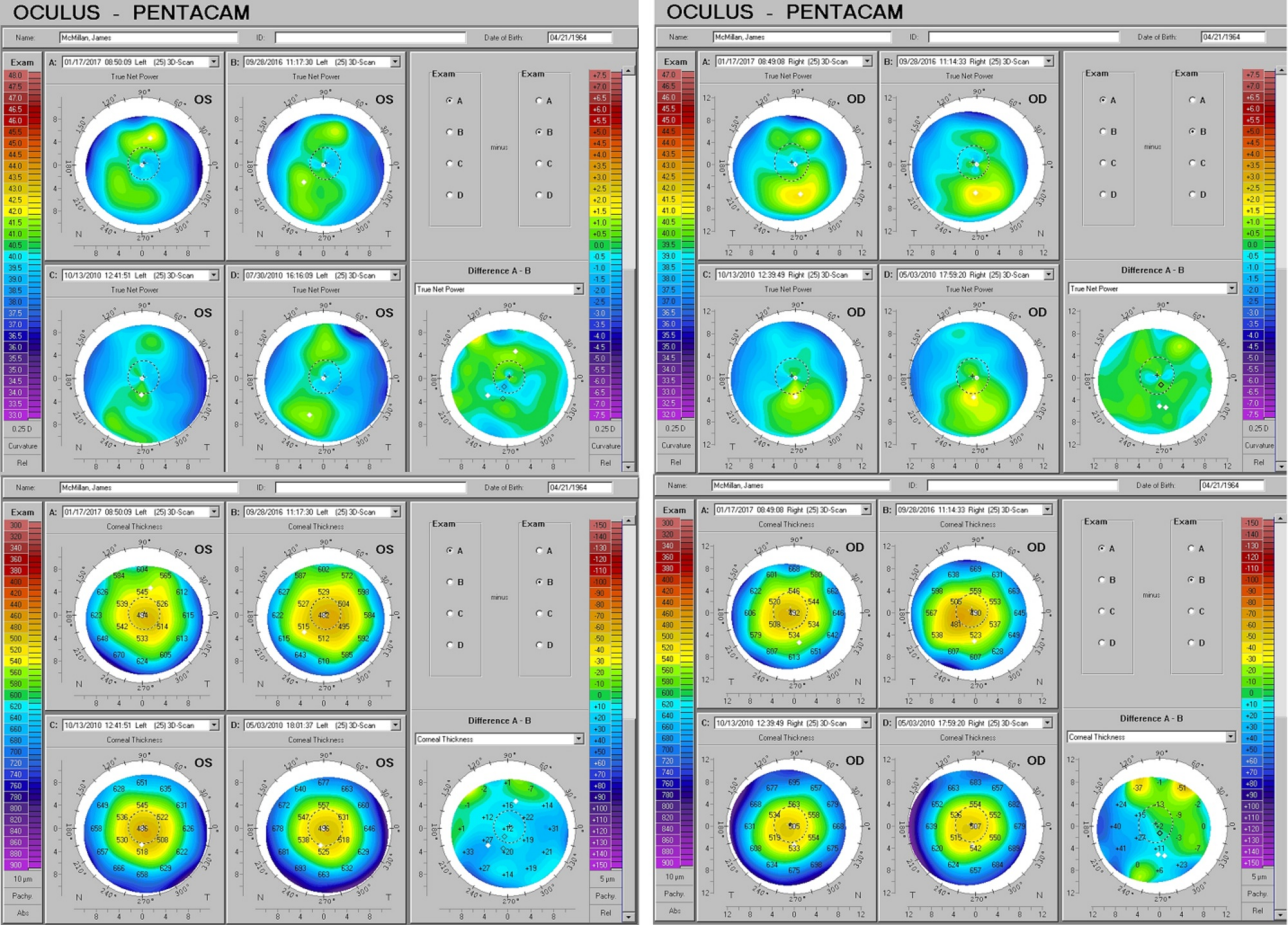
Dose-related Response to D3 Supplementation Corneal form and optical quality improved under higher dose D3 (10,000 IU/day), relapsed at lower dose (5000 IU/day), and improved again with the resumption of 10,000 IU/day.

A 52-year-old ophthalmologist—the author/investigator of this report—was found to have thin corneas and irregular astigmatism in 2010. A rapid improvement was appreciated at 10,000 IU per day over the first three months of therapy. D3 intake was subsequently reduced to 5,000 IU per day for the ensuing six years, maintaining the serum 25(OH)D3 at 70-75 ng/cc (assessed annually) but, surprisingly, demonstrating a marked relapse in both thinning and irregular power distribution, when remapped with the Pentacam in late 2016. Vitamin D was restored to 10,000 IU/day and, in less than three months, an overt improvement in corneal shape, optics, and thickness were again readily apparent (Figure 9).

## Discussion

**Figure 10:**
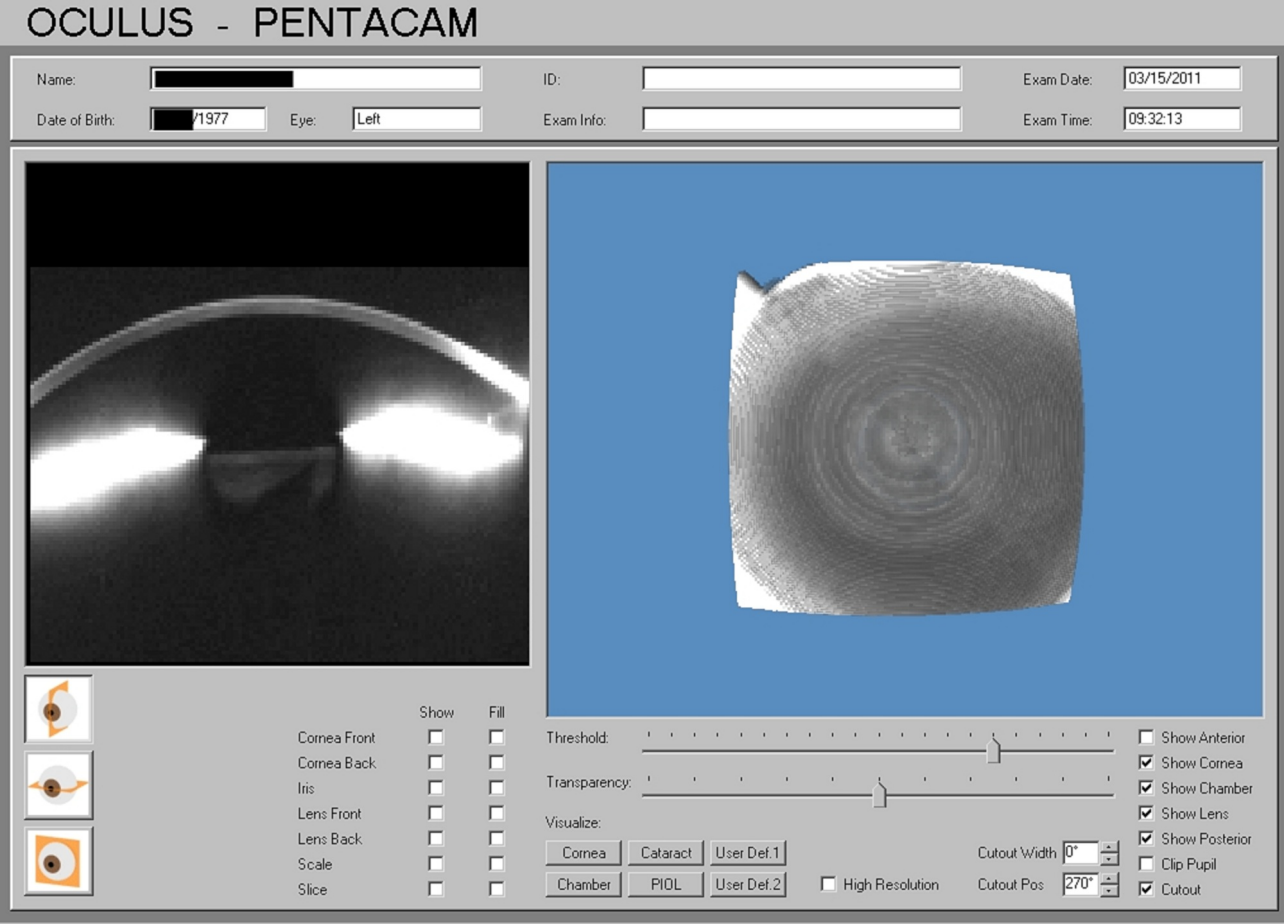
Typical Low D3 Nebula Common finding of a central nebula with chronic low D3, which resolves with adequate supplementation.

The predictable presence of two findings is readily found in a chronically vitamin D deficient patient using the Oculus GmbH Pentacam. The first is a central, disc-like region of faint corneal stromal haze, an example of which is shown in Figure 10. This haze is generally not well visualized at the slit lamp, though in the most prominent cases (typically in elderly individuals), it can be discerned with indirect illumination. Cross-sectional imaging by the Pentacam suggests it extends through the full thickness of the corneal stroma in a relatively uniform fashion. The haze clears progressively with D3 supplementation.

**Figure 11:**
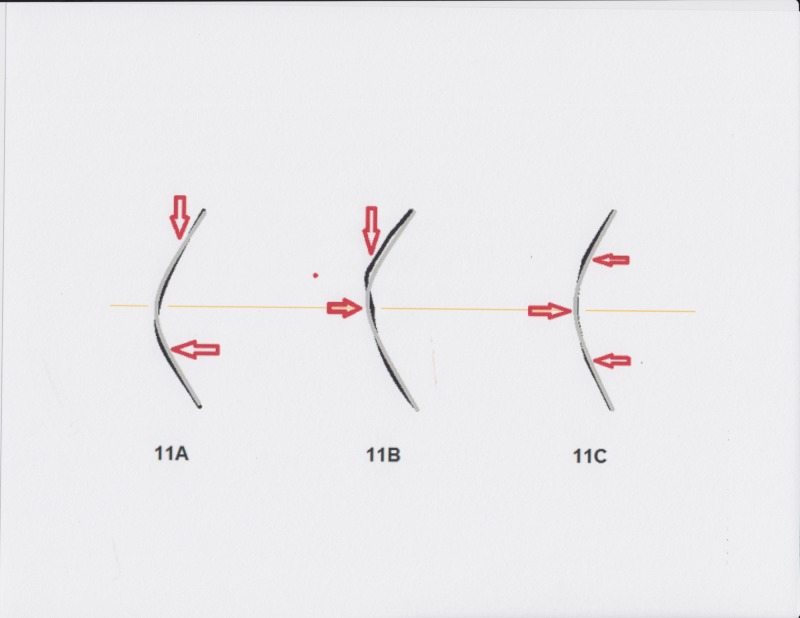
Corneal Mechanical Deformation Patterns Illustration of the three most commonly encountered distortion patterns associated with low D3 and representing variations of the mechanical arch compression response. A) Superior depression by the upper eyelid leading to out-bulging and steepening of the inferior cornea. B) Superior depression creating a more immediate "ripple" and wave pattern beneath, often seen in thicker-than-average corneas. C) Apical compression with the resulting steepening of the immediate surround, possibly caused by external central compression during Bell's reflex.

The second finding is irregular astigmatism, which tends to take one of three predictable forms, all of which represent deformation effects upon the mechanics of an arch. In the most common version, mostly seen in corneas under 560 um center thickness, a “tear-drop” pattern of relative inferior steepening forms. This is consistent with a direct depression of the superior cornea via the weight and elastic compression exerted by the upper eyelid, depicted in Figure 11A. This superior depression (which the Pentacam can quantitate and compare in contrast to an idealized model) drives a compensating elevation below the center, creating a wave of asymmetry across the visual axis for which there is no regular optical solution. In addition, the usually “almond-shaped” arched configuration of the upper eyelid leads to a disproportionate vector of this force being exerted inferonasally in the majority of people. This is seen in extreme cases with the pronounced inferonasal steep displacement characteristic of keratoconus. Opposing these upper lid compressive forces are globe/corneal mechanical properties: elasticity, “dome mechanics” with respect to the distribution of applied forces, thickness and also the intraocular pressure, all of which would otherwise contribute to forming an ideal aspheric shape (given the normal corneal thickness tapering toward its axial apex). In keeping, variations in local corneal thickness, pathology (scars, pterygia, dehydration and Dellen formation), alteration of elasticity/compliance (keratotomy effects, cross-linking, etc.), upper eyelid position (ptosis, retraction) and lid thickness and weight (chalazia, chronic blepharitis, hemangioma, dermatochalasis, etc.) are seen to impact and alter corneal shape disturbance in predictable ways. A second deformation pattern is more common in corneas thicker than 560 um. This pattern shows a primary “ripple” immediately below the usual upper eyelid margin's resting position in primary gaze, just above the superior pupil margin in room light. There is commonly an “echo” of additional diminishing waves inferior to it. This pattern is illustrated in Figure 11B. The third easily identifiable pattern is pronounced apical flattening as shown in Figure 11C. This is frequently encountered in hyperopic individuals and may stem from the particular effect of Bell’s phenomenon: the underside of the upper eyelid and orbital roof contacting and compressing the up-turned cornea at night. Combinations featuring any mix of the three patterns are also readily encountered.

Adequate D3, amazingly without fail, leads to steady reversal of any of these distortions in 100% of cases observed. Regions of excessively steep power drop and the low regions rise, producing more uniform optics. Irregular astigmatism reduces first but, generally, the regular component begins to decrease as well. Curiously, if at any age there is some degree of lenticular/internal astigmatism, the corneal component approaches a 90 degree-opposite counter-balancing value, closing upon a net spherical (non-astigmatic) outcome. This implies a feedback-driven, self-adjustment capability may remain in effect even later in life.

Consistent with limbal stem cell location and peripheral initiation of growth, the changes in shape, mechanical properties and even changes in thickness appear to initiate at the periphery and spread centrally. These shifts often exhibit properties of a moving wave, generating a trough preceding the wave peak as it converges upon the corneal center. As with a wave entering the shallower water near a beach, the peak steep zone may even rise, focally steepening further, as it moves into successively thinner cornea. The waveform then “breaks” and dissipates, settling into a more idealized aspheric form. The dramatic speed with which restoration of an optimal serum 25(OH)D3 level achieves objective response suggests improved mechanical properties are initially a physiologic phenomenon as opposed to structural synthesis, although the latter appears to follow later and cement the improvement. This is given weight by the enhanced effect seen via the addition of a topical corticosteroid. The latter perhaps represents a mineral-corticoid (rather than glucocorticoid) effect, with the resulting shift in the water/salt balance bringing about a rapid alteration in the stromal biomechanics. Overall, the improved optics of a vitamin D-replete state appear to result from enhanced corneal mechanical properties reliably yielding an optimized arch/dome “engineering” response. 

The implications regarding D3 influence upon intraocular pressure are highly intriguing. Aqueous outflow would logically be impacted by changes in limbal-region mechanical properties, but the control of internal fluid pressure may be implicated in producing some of those same mechanical changes as well. Pressure changes might thus not be always just a response to shifting mechanical properties of the globe, but may, in fact, be a major agent in creating them. Aqueous pressure exerts a direct effect upon the shape of the cornea and globe as an “inflating” force, but it also has a hydrostatic impact upon these structures (mediated by the corneal endothelium, in particular). It influences hysteresis and the nature of the mechanical response to those shaping forces. This combination is rich with potential for a feedback-driven process. Might the intraocular pressure be an instrument for modification and/or stabilization of the eye’s optics? In the pathological state—for instance, pediatric glaucoma—that relationship is overt in its negative consequences, with the more compliant globe delivering dramatic disc cupping and axial elongation, but not necessarily an elevated intraocular pressure measurement. Seen in this light, glaucoma may represent derangement of a process charged primarily with optical stability and/or adaptation to adverse optical disturbance. The aqueous would go beyond a “fill, remove and replenish” function to act as an instrument for globe modification: actively “inflating/deflating”—albeit very slowly—according to feedback-generated need. Under normal circumstances, this would support refining the optical configuration in concert with the elastic properties of the cornea and globe as a whole. In pathological states, peril becomes compounded.

Implications for emmetropization and myopia

Given its well-known impact on bone development and connection to sunlight, a role for vitamin D was the logical first choice for explaining the long-observed inverse association between daylight exposure and incidence of myopia. While that association has remained solid through multiple studies, the role of D3 as the agent of causation has been controversial from early on, due to findings of only small or even no significant differences in the blood levels between myopes and non-myopes [1,4]. Unfortunately, it does not appear any controlled prospective trial has yet been done to assess the impact of effective supplementation, though a non-controlled prospective trial in a small number of patients was reported by Knapp as far back as 1939 [3]. Population cross-sectional studies do not address the possibility—the likely explanation in the author’s opinion—that most young people are chronically deficient under present-day circumstances, even at lower latitudes and in sunny regions, owing to a multitude of factors (indoor schooling, clothing, lack of substantial vitamin D sources in the diet, pollution, sunblocks, skin pigmentation/tan, etc.), but some are more susceptible to that state than others. The latter would represent the “hereditary” element of myopia tendency. The findings herein reported suggest rather an indirect response to D3 deficiency-induced corneal distortion actually leads to myopic shift and subsequent progression, ironically due to optically driven feedback mechanisms normally meant to foster emmetropia.

As described, the lack of sufficient vitamin D reliably produces substantial irregular astigmatic states, most of which directly involve the central optical zone. This brings about a situation wherein the eye cannot be adequately in uniform focus under any conditions. Evolving understanding of the process of emmetropization in chicks includes a response weighted according to the spatial ratio of retina receiving myopic versus hyperopic defocus [13]. All three corneal distortion patterns linked to insufficient vitamin D status described earlier create relative flattening within significant regions of the optical zone—yielding hyperopic defocus—yet also show localized steepening below or immediately adjacent, those regions featuring myopic defocus. This optical “turbulence” results in a “perfect storm” at that point, with regions of hyperopic defocus alongside regions of myopic defocus. Correction of the latter exacerbates the relative influence of the former—a vicious cycle. In addition, larger pupil size enlarges the area of irregular defocus and longer wavelength illumination will relatively dominate central retinal stimulation, longer wavelength light being less susceptible to deviation via irregular optics than wavelengths at the shorter end of the spectrum. It has been shown that longitudinal chromatic aberration provides a mechanism for sensing hyperopic vs. myopic defocus via changes in luminance and chromatic contrast [13-14], so the resulting dominance of longer wavelength light upon the central retina would all the more drive a “corrective” growth response toward axial lengthening and resultant myopia. Add to that a common preference for “warmer” lighting indoors—weighted toward the longer wavelengths as well—and it becomes no surprise asymmetrically defocused, growing eyes lean toward axial elongation and myopic outcomes. Eyeglass correction cannot address this imbalance, either, offering a rigidly limited two-dimensional solution to what is very much a three-dimensional problem. For the most part, soft contact lenses echo corneal distortion to some degree and uniformity of focus will then suffer from the same conflicts. A rigid contact lens might be seen to offer at least a partial solution to this conundrum and relative suppression of myopic progression has been reported accordingly [15], but their well-known tendency to impose apical flattening and corneal irregularity, as well as their inability to mask refractive aberrations of the inner corneal surface, tear lens and net corneal refractive power frustrate the benefit, also an observed phenomenon [15].

Applying the understanding of this concept clinically in the herein reported patient population has nearly eradicated return visits for “unhappy refractions” and allowed effective trouble-shooting for second opinions sought for this complaint. Usual refraction technique, in particular, the application of the Jackson cross-cylinder in the determination of cylinder power, appears to innately favor over-correction of the focal elevations of corneal power at the expense of the low regions. In some cases, a single “clock-hour “ region of the optical zone may be critically in focus on a 20/20 line, while 11 “clock-hours” are over-corrected. It also drives higher-than-necessary cylinder correction, in particular, unless one specifically rounds downward while maintaining spherical equivalence. Respecting patients’ subjective response to the maneuver, in most cases, will prove they prefer the lower cylinder correction, which corresponds to the majority of the optical zone’s needs, rather than supports a focally steep region of maximum demand. The patient might comment the image isn’t “crisp,” but it proves more comfortable/tolerable/desirable in general use.

This explains the young high school student, who can target a friend with a ball at 50 meters, but complains of difficulty reading the “smart board” in class despite manifesting near-plano refraction. The irregular cylinder component is not picked up fully (or sometimes at all) by manifest refraction: the mix of flat and steep regions along the same meridian cancel one another out to a degree, while still yielding a net loss of resolution. Lower light and contrast conditions, illuminated targets, pixels on screens and reading, in particular, are much more prone to negative impact from this irregular astigmatic distortion and defocus, due to the near-axial location of the deviations, accommodation-driven pupillary constriction converging further upon them, and probably elements of constructive and destructive optical interference. Topographers lacking ability to directly assess the central optical zone, including the majority of placido-disc systems long in use, may under-calculate the degree of aberration or even miss its presence entirely. The use of the Oculus Pentacam has proven invaluable in identifying this phenomenon.

Why the “disc” of central stromal haze forms in the setting of inadequate vitamin D availability is unknown, but it reliably clears with effective supplementation. Its localized nature appears to be a physical phenomenon and not just an artifact of the plane of imaging illumination relative to that of the corneal curvature. Whether there is any connection to “stromal microdots” revealed by in vivo confocal microscopy in many adult corneas and reported prone to increase with age is an interesting question [16]. The ability of the Pentacam to reproducibly quantify the reflectance measurement and follow this parameter objectively as it decreases (or increases in those still vitamin D deficient) has proven helpful in documenting the response to vitamin D replacement and allows correlation of reflectance values directly with clinical symptoms. Many individuals will report images lack sharp definition when reflectivity values measure above 20 units in the axial mid-stroma. Those in excess of 25 units will frequently complain of glare. The latter is particularly evident in older, life-long residents of the Pacific Northwest who have had cataract surgery already. Their corneas appear within the range of normal clarity at the slit lamp, typically with a well-centered intraocular lens and no capsular concerns. Symptoms otherwise seem incongruous with the exam findings, but make sense in light of the identified haze. In some cases, specifically hunting for the haze, it can be appreciated clinically with a brighter, broad slit beam oriented obliquely through the stroma. 

Glaucoma

A potential role of vitamin D with regard to glaucoma is not novel, but there has been little definition of that role to date; sporadic literature on the subject spans more than 6 decades [17-21]. These new findings can provide synergistic insight, however, into recent work exploring corneal biomechanical properties for differentiating normal from glaucomatous eyes [22] and genetic associations linking central corneal thickness, keratoconus and open-angle glaucoma [23]. The latter include the already vitamin D-associated FOXO1 locus [24]. The present case data, supporting a direct role in significant improvement of intraocular pressure control with adequate supplementation, taken in concert with the clearly demonstrable structural changes brought about by D3 availability (as well as the suggestion of an additional capability to suppress the “steroid effect” upon intraocular pressure), hopefully will enkindle additional research. Of note, newly diagnosed glaucoma has largely ceased to present within this patient population under study over the last ten years, a phenomenon otherwise difficult to explain.

Dry Eyes

The ability to control dry eye symptoms and findings with adequate availability of D3 has proven tremendously beneficial, in particular, with the synergistic application of a topical steroid, ideally non-preserved. Topical steroids have long been known to improve dry-eye associated symptoms, findings, and inflammation. However, their long-term use is restricted by the risk of adverse effects like cataract formation and intraocular pressure rise. In contrast, in the presence of adequate D3, valuable improvement is seen in the great majority of patients with short-term and low dose topical steroid application for which risks are minimal. In the study population, approximately 80% report total or near-total remission of symptoms (anecdotal). About 20% do not respond as well, reflecting the multi-factorial nature of the condition; however, even those who earlier “failed” topical cyclosporine therapy may achieve relief by trying it again in concert with optimal vitamin D replacement in progress (anecdotal). Vitamin D’s possible influence and usefulness in addressing dry eye disease have been previously proposed and vitamin D deficiency has been linked to dry eye disease [2-9], but, to date, no specific protocol has been advanced recognizing documented success via controlled prospective studies in humans. Topical application of vitamin D formulations for treatment of dry eyes and ocular surface disease was the subject of a series of patents filed by Kita (2000) and Itoh et al (1991-2002) [5], but they only provided limited observations from a small series of animal studies in support of the claims. More recently, lifitegrast (XiidraTM), a Lymphocyte Function Associated Antigen 1 (LFA-1) binding agent has been introduced for treatment of dry eye. This compound is believed to interfere with the binding of T cells to the Intercellular Adhesion Molecule 1 (ICAM-1) and suppress pro-inflammatory cytokine production involved in the pathogenesis of an ocular surface disease. Significantly, D3 interacts with both ICAM-1 and LFA-1 to modulate immune function [25-26] and supplementation may offer a synergistic, augmented or adjunctive benefit in concert.

That vitamin D forms show synergy with topical steroids for an ocular surface disease is consistent with the enhanced therapeutic effect already established for psoriasis—another vexing, chronic, and surface-located inflammatory condition—achieved using just such a combination [27]. The findings in the present study will hopefully fuel even more investigation and perhaps lead to the development of topical steroid-vitamin D analogue preparations with a variety of clinical applications for the eye and other organ systems. If suppression of steroid-response intraocular pressure rise can be consistently demonstrated in follow-up controlled studies, the combination would be all the more encouraging to employ.

Keratoconus

Within this evolving paradigm, keratoconus may be uniquely explained and understood as lying at one extreme of a bell-shaped curve of corneal response to chronically low D3. Given the pattern of occurrence and the better response observed to higher supplementation, this previously confounding disease can be reasonably described as the ocular version of “vitamin D-resistant rickets.” This interpretation readily explains the recurrence of keratoconus after corneal grafting, too, since the patient, in all likelihood, remains in the same or even a worsening state of vitamin D deficiency with increasing age.

Remarkably, the ability to achieve improvement and even reverse keratoconus findings utilizing forms of vitamin D supplementation was also previously reported by Knapp in 1938 [28]. Lacking the benefit of computerized topography and structural analysis, he meticulously created life-casts of keratoconus-afflicted corneas. With the help of the New York University engineering and physics departments providing micrometer measurements, he demonstrated the flattening of the cones in all six of the subjects available for follow-up after six months of increased vitamin D supplementation. He was also able to show an overall decrease in corneal height in at least five of the six. No further progress based upon his findings appears to have been made until now. The re-discovery of keratoconus reversal with adequate D3 reported here was arrived at without any knowledge of Knapp’s work. His publications were uncovered during a subsequent search and review of historical literature.

For keratoconus, collagen cross-linking techniques have achieved a beneficial degree of stabilization. Also, the asymmetric implantation of intrastromal ring-segments may improve the optical properties of keratoconic eyes and impede the structural decline when combined with cross-linking [29]. Seen in the light of the vitamin D deficiency-related biomechanical model discussed in this report, the improved outcome with the single segment, asymmetrical, stromal implant placement described by Sharma and Boxer Wachler is readily appreciated, specifically reinforcing the weakened cornea at the location needed to support upper eyelid weight. By contrast, optical improvement is likewise seen following upper eyelid blepharoplasty, repair of significant ptosis, or in relief of substantial dermatochalasis, via reducing the deformational mass instead of reinforcing the supporting corneal structure that resists it.

Cataracts

With regard to cataracts, the demand for surgery in the studied population declined precipitously over the last 10 years, from approximately five cases per month (reflecting a small, solo practice scheduling seven to 10 patients daily) to one every three to four months. Patient demographics did not change over the time period in question and most other ophthalmic practices in our area have experienced a contemporary rise in demand for cataract surgical services (anecdotal), consistent with national trends and the aging of the “Baby Boom” generation. The only factor known to have changed in the studied population is increasing compliance with recommended D3 supplementation. Nuclear density, rated via Pentacam reflectivity, appears stabilized or even reduced in some (anecdotal). However, the combination of increasing irregular axial corneal astigmatism in the D3 deficient population, when added to concurrent cataract development, readily produces intrusive symptoms. Resolving the corneal distortion thus allows the symptoms to diminish to the point of toleration, delaying the need for surgery. While not eliminating the eventual need for cataract extraction, a significant delay in the need for surgery could offer benefits on a global scale.

Macular degeneration

A protective role for vitamin D is likewise emerging for macular degeneration [1]. The observations herein reported offer additional insight, though, into a peculiar phenomenon long observed by the author: near-universal association between macular degeneration and substantial against-the-rule astigmatism. Given that the term “against the rule” (ATR) describes the vertical axis refractive cylinder being less commonly encountered than the horizontal axis “with the rule” astigmatism in the normal population, the curiosity that a great majority of AMD-afflicted individuals exhibit ATR astigmatism—even after otherwise successful cataract surgery should have eliminated any lens/nuclear ATR component—suggests the two conditions are closely linked in some fashion, perhaps by an element of underlying vitamin D deficiency.

The vitamin D deficiency distortion patterns described tend to induce corneal ATR cylinder effects, the full magnitude of which may escape detection without the assistance of accurate topographical assessment. In the setting of significant irregularity, manifest refraction may not even be able to provide full correction, and the resulting acuity discrepancy is then too logically ascribed entirely to the macular disease. In the studied population, there have been more than a few individuals who regained their ability to read common text or even drive once the corneal shape was improved and the stromal haze reduced with adequate vitamin D supplementation, even though there was no other remarkable change in macular appearance, drusen, or pigment epithelial changes (anecdotal). Older individuals are logically at a particularly high risk for vitamin D deficiency, frequently experiencing more limited time outdoors, employing protective strategies against skin cancer, and, in the Pacific Northwest, simply having lived longer in a darker part of the world. The commonplace magnesium deficiency resulting from over-farming and soil depletion further compounds the problem. Serial retinal Optomap (Optos Inc., Dunfermline, Scotland) images from AMD patients adequately supplemented with D3 support the possibility that increased availability may be associated with diminished drusen, stability of pigment epithelial changes, and, possibly, even arrested geographic atrophy (anecdotal). These observations await further exploration and confirmation.

Vitamin D supplementation

A critical observation is that beneficial responses are usually only realized when the serum 25(OH)D3 level rises above 50 ng/cc and optimal response begins around 70-80 ng/cc. Historically, laboratories referenced a normal range of 30 to 100 ng/cc, so, in that respect, the apparent ideal coincides with the middle of the normal range—ordinarily a desirable thing. In keeping, levels above 50 ng/cc are found in contemporary populations with significant daily sun exposure [30].

By contrast, in the author’s experience, the majority of inadequately supplemented residents of Western Washington State have 25(OH)D3 levels well below 30 ng/cc. A recent study suggests levels under 30 ng/cc are likewise prevalent throughout the USA, even at lower latitudes, with theoretical access to adequate sunshine [12], reflecting population trends toward predominantly indoor employment and education, sun-protective clothing, sunblocks, air pollution, etc. Faced with such a baseline, to achieve serum 25(OH)D3 greater than 50 ng/cc, local experience has been that most adults require vitamin D3 supplementation somewhere between 5,000 and 10,000 IU/day and a small number have needed 15,000 IU/day. Children have been found to require approximately 1000 IU/25 pounds (ll.3 kilograms) body weight/day. These are substantially above the currently recommended dose of 600-800 IU/day (and the “safe upper limit” of 4000 IU/day) advocated by the Institute of Medicine since 2011 but consistent with recommendations from the Endocrine Society [30]. An additional concern is that the responses and improvements described have not yet been observed in the studied population via supplementation by ergocalciferol (vitamin D2), even at very high doses and in prescription formulations. Given that ergocalciferol is by far the most common food-additive and fortification analogue of vitamin D, and the most common form available by prescription in the United States, the lack of a response in the cornea—despite proving 100 % predictable in the case of cholecalciferol/D3—raises questions about the physiologic efficacy of vitamin D2 in many regards. Knapp did report a response to ergocalciferol/D2 in his 1938 keratoconus research, but it was given at an extremely high dose by comparison, up to 50,000 IU daily. Interestingly, in the author’s experience, 5,000 IU/day of cholecalciferol/D3 quickly normalized and stabilized serum calcium levels of a patient with presumed secondary hypoparathyroidism, who had been consistently hypocalcemic for years while taking prescription ergocalciferol at 50,000 IU every other day. This phenomenon would likewise benefit from further investigation.

A major limitation of this report is the challenge of evoking the abundance of topographical data obtained over a decade in a concise form for publication and review. While some parameters determined by the Pentacam may lend themselves to a tabular or graphical presentation, the overall consistency of the eye's response to adequate D3 is best appreciated by a serial evaluation of multiple aspects in concert. For instance, in some with keratoconus, posterior elevation may show a reduction and average corneal thickness a desirable increase, yet the minimum internal radius may transiently decrease as the inferior displacement of the maximum curvature shifts/restores back toward the corneal axis. By contrast, in others, the internal radius can increase without an initial change in thickness or a significant change in measured posterior elevation. Both patterns contribute to an optical improvement and represent the reversal of an aspect of disease progression; yet, in combination, the data would, to some extent, mathematically conflict. The latter can be resolved with adequate numbers and time, such as may be afforded by a controlled trial in a corneal specialty clinic. In addition, there are many potentially confounding variables inherent in uncontrolled observations during clinical practice: compliance, diet, sun exposure, use of sunblocks, dietary presence or absence of cofactors, and coexistent pathophysiology to name just a few. While only a handful of illustrative examples can be presented here, they nevertheless represent a multitude of similar cases for which documentation does exist, driving an imperative to inform, and hopefully inspire, controlled trials.

## Conclusions

The adequate replacement of vitamin D3 in the population under evaluation has resulted in the arrest and reversal of keratoconus and myopic progression and, in addition, suggests a promising, beneficial impact upon dry eye, glaucoma, cataract, and macular degeneration concerns. Unlike many conditions under scientific study, the response is fast and, so far, has been amazingly predictable. The ability to prevent the onset of myopia may even prove absolute. It is hoped that the wide applicability of these insights will rapidly encourage controlled studies to confirm, explore, and further delineate and extend upon these findings.

## References

[1] Jee D, Kang S, Yuan C, Cho E, Arroyo JG (2016). Serum 25-hydroxyvitamin D levels and dry eye syndrome: differential effects of vitamin D on ocular diseases. PLoS One.

[2] Ito S (2017). Ophthalmic composition containing active vitamin D. USPTO.

[3] Knapp AA (1939). Vitamin-D complex in progressive myopia. Am J Ophthalmol.

[4] Mutti DO, Marks AR (2011). Blood levels of vitamin D in teens and young adults with myopia. Optom Vis Sci.

[5] Pan CW, Qian DJ, Saw SM (2017). Time outdoors, blood vitamin D status and myopia: a review. Photochem Photobiol Sci.

[6] Williams KM, Bentham GCG, Young IS (2017). Association between myopia, ultraviolet B radiation exposure, serum vitamin D concentrations, and genetic polymorphisms in vitamin D metabolic pathways in a multicountry European study. JAMA Ophthalmol.

[7] Alsalem JA, Patel D, Susarla R (2014). Characterization of vitamin D production by human ocular barrier cells. Invest Ophthalmol Vis Sci.

[8] Mutti DO, Cooper ME, Dragan E (2011). Vitamin D receptor (VDR) and group-specific component (GC, vitamin D-binding protein) polymorphisms in myopia. Invest Ophthalmol Vis Sci.

[9] Annamaneni S, Bindu CH, Reddy KP, Vishnupriya S (2011). Association of vitamin D receptor gene start codon (Fok1) polymorphism with high myopia. Oman J Ophthalmol.

[10] Sanctis UD, Missolungi A, Mutani B, Richiardi L, Grignolo FM (2007). Reproducibility and repeatability of central corneal thickness measurement in keratoconus using the rotating scheimpflug camera and ultrasound pachymetry. Am J Ophthalmol.

[11] (2017). Top 101 cities with the lowest average sunshine amount (population 50,000). Available at: http://www.city-data.com/top2/c475.html.

[12] Schleicher RL, Sternberg MR, Lacher DA (2016). The vitamin D status of the US population from 1988 to 2010 using standardized serum concentrations of 25-hydroxyvitamin D shows recent modest increases. Am J Clin Nutr.

[13] Tse DY, To CH (2011). Graded competing regional myopic and hyperopic defocus produce summated emmetropization set points in chick. Invest Ophthalmol Vis Sci.

[14] Rucker FJ, Wallman J (2012). Chicks use changes in luminance and chromatic contrast as indicators of the sign of defocus. J Vis.

[15] Walline JJ, Jones LA, Mutti DO, Zadnik K (2004). A randomized trial of the effects of rigid contact lenses on myopia progression. Arch Ophthalmol.

[16] Hillenaar T, Cleynenbreugel HV, Remeijer L (2012). How normal is the transparent cornea? Effects of aging on corneal morphology. Ophthalmology.

[17] Guist G, Steffen C (1953). Application and mechanism of high dosage of vitamin D therapy of glaucoma [Article in German]. Klin Monbl Augenheilkd Augenarztl Fortbild.

[18] Kaushik S, Pandav SS, Banger A, Aggarwal K, Gupta A (2012). Relationship between corneal biomechanical properties, central corneal thickness, and intraocular pressure across the spectrum of glaucoma. Am J Ophthalmol.

[19] Kutuzova GD, Gabelt BAT, Kiland JA, Hennes-Beann EA, Kaufman PL, DeLuca HF (2012). 1α,25-Dihydroxyvitamin D3 and its analog, 2-methylene-19-nor-(20S)-1α,25-dihydroxyvitamin D3 (2MD), suppress intraocular pressure in non-human primates. Arch Biochem Biophys.

[20] Krefting EA, Jorde R, Christoffersen T, Grimnes G (2014). Vitamin D and intraocular pressure-results from a case-control and an intervention study. Acta Ophthalmol.

[21] Goncalves A, Milea D, Gohier P (2015). Serum vitamin D status is associated with the presence but not the severity of primary open angle glaucoma. Maturitas.

[22] Lee R, Chang RT, Wong IY, Lai JS, Lee JW, Singh K (2016). Novel parameter of corneal biomechanics that differentiate normals from glaucoma. J Glaucoma.

[23] Lu Y, Vitart V, Burdon KP (2013). Genome-wide association analyses identify multiple loci associated with central corneal thickness and keratoconus. Nat Genet.

[24] Chen S, Villalta SA, Agrawal DK (2016). FOXO1 mediates vitamin D deficiency-induced insulin resistance in skeletal muscle. J Bone Miner Res.

[25] Matsuzaki J, Tsuji T, Zhang Y (2006). 1alpha,25-dihydroxyvitamin D3 downmodulates the functional differentiation of Th1 cytokine-conditioned bone marrow-derived dendritic cells beneficial for cytotoxic T lymphocyte generation. Cancer Sci.

[26] Martinesi M, Treves C, Dʼalbasio G, Bagnoli S, Bonanomi AG, Stio M (2008). Vitamin D derivatives induce apoptosis and downregulate ICAM-1 levels in peripheral blood mononuclear cells of inflammatory bowel disease patients. Inflamm Bowel Dis.

[27] Augustin M, Mrowietz U, Bonnekoh B (2014). Topical long-term therapy of psoriasis with vitamin D3 analogues, corticosteroids and their two compound formulations: position paper on evidence and use in daily practice. J Dtsch Dermatol Ges.

[28] Knapp AA (1939). Vitamin-D-complex treatment of keratoconus. Am J Ophthalmol.

[29] Sharma M, Wachler BSB (2006). Comparison of single-segment and double-segment intacs for keratoconus and post-LASIK ectasia. Am J Ophthalmol.

[30] Holick MF, Binkley NC, Bischoff-Ferrari HA (2011). Evaluation, treatment, and prevention of vitamin D deficiency: an Endocrine Society clinical practice guideline. J Clin Endocrinol Metab.

